# A critical review of the current knowledge regarding the biological impact of nanocellulose

**DOI:** 10.1186/s12951-016-0230-9

**Published:** 2016-12-01

**Authors:** C. Endes, S. Camarero-Espinosa, S. Mueller, E. J. Foster, A. Petri-Fink, B. Rothen-Rutishauser, C. Weder, M. J. D. Clift

**Affiliations:** 1 Adolphe Merkle Institute, University of Fribourg, Chemin des Verdiers 4, 1700 Fribourg, Switzerland; 2 Australian Institute for Bioengineering and Nanotechnology (AIBN), Cnr College Rd & Cooper Rd, Building 75, Brisbane, QLD 4072 Australia; 3 Department of Materials Science and Engineering, Macromolecules Innovation Institute (MII), Virginia Polytechnic Institute and State University (Virginia Tech), 213 Holden Hall, 445 Old Turner Street, Blacksburg, VA 24061, USA; 4 In Vitro Toxicology Group, Swansea University Medical School, Singleton Park Campus, Swansea, SA2 8PP Wales, UK

**Keywords:** Nanocellulose, Cellulose nanocrystals, Human health, Risk, Exposure, Hazard, Nanofibers, Nano-object-cell interactions, Nanotoxicology

## Abstract

Several forms of nanocellulose, notably cellulose nanocrystals and nanofibrillated cellulose, exhibit attractive property matrices and are potentially useful for a large number of industrial applications. These include the paper and cardboard industry, use as reinforcing filler in polymer composites, basis for low-density foams, additive in adhesives and paints, as well as a wide variety of food, hygiene, cosmetic, and medical products. Although the commercial exploitation of nanocellulose has already commenced, little is known as to the potential biological impact of nanocellulose, particularly in its raw form. This review provides a comprehensive and critical review of the current state of knowledge of nanocellulose in this format. Overall, the data seems to suggest that when investigated under realistic doses and exposure scenarios, nanocellulose has a limited associated toxic potential, albeit certain forms of nanocellulose can be associated with more hazardous biological behavior due to their specific physical characteristics.

## Background

Since the emergence of nanotechnology as a field in its own right, a continuously increasing number of new nanomaterials have been developed, which are potentially useful for applications that range from healthcare products to high-performance engineering materials [1–3]. Several forms of nanocellulose, in their raw format, have been demonstrated to exhibit attractive property matrices and are potentially useful for the paper industry, as a reinforcing filler in polymer composites, basis for low-density foams, in packaging materials, additive in colloidal systems such as adhesives and paints, zero-calorie filler/thickener/stabilizer in a wide variety of food products, and in hygiene, cosmetic, and medical products [4, 5]. Although (microcrystalline) cellulose has long been used in healthcare products such as wound healing tissue and dialysis membranes, as well as a food additive, little is known as to the potential adverse biological impact of its nanoscale variants, whose commercial exploitation only begun in the last few years [6, 7].

**Fig. 1:**
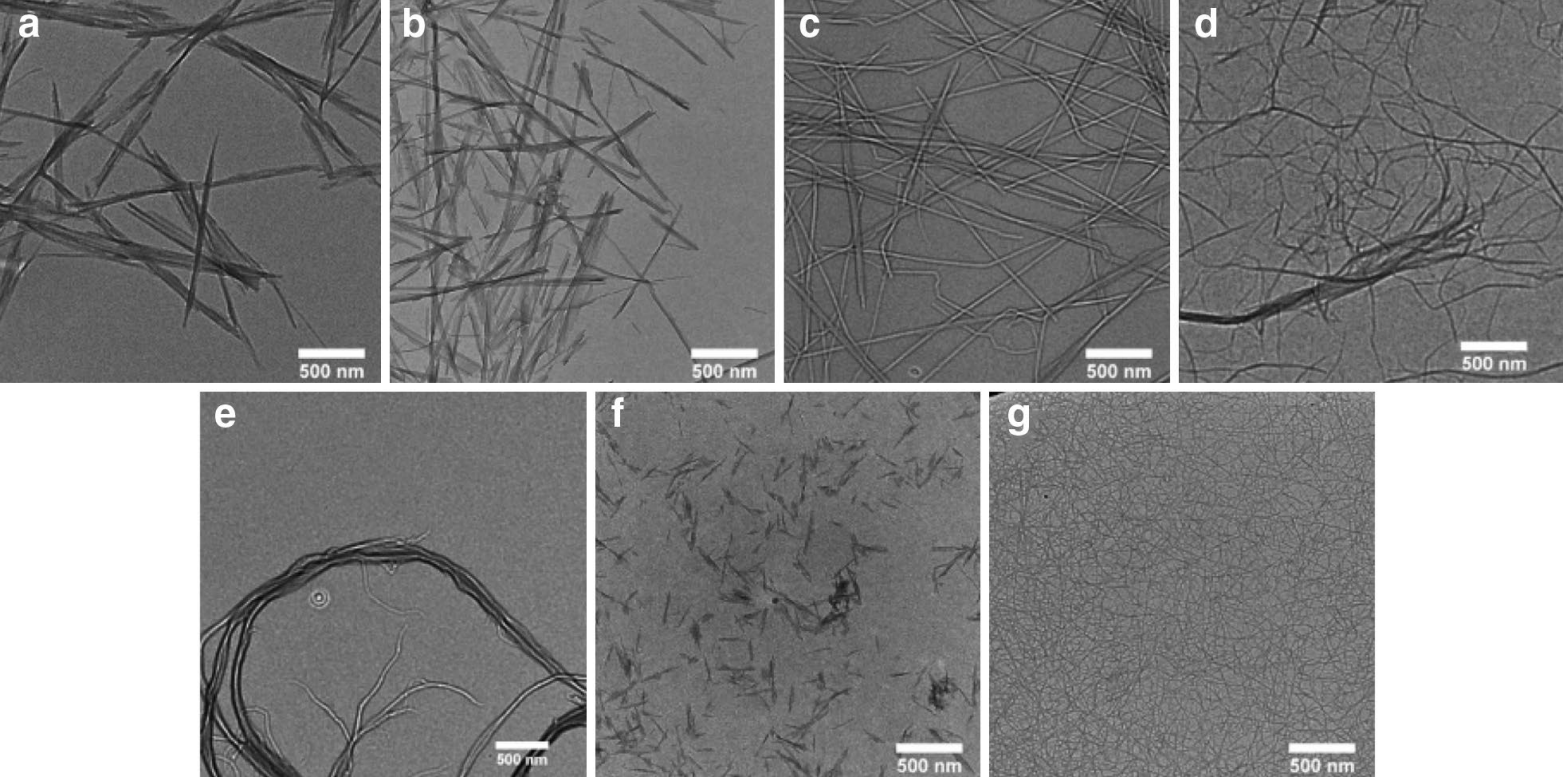
Transmission electron microscopy images of selected nanocellulose types. CNCs isolated by HCl (**a**) and H_2_SO_4_ hydrolysis (**b**) from bacterial cellulose, H_2_SO_4_ hydrolysis from tunicate mantles (**c**) or wood pulp (**f**) and nanofibrillated cellulose obtained by enzymatic (**d**), mechanical (**e**), or 2,2,6,6-tetramethylpiperidinyl-1-oxyl (TEMPO) mediated oxidative (**g**) degradation of wood pulp. The figure is reprinted with permission from Sacui et al. [96] © (2014) American Chemical Society

Cellulose, the most abundant polymer in the world, is found in plant cell-walls, certain sea creatures, *e.g.* tunicates, and algae, *e.g. Valonia*. It is also produced by several bacteria such as *Acetobacter xylinum* [8–11]. Cellulose is a carbohydrate, whose repeat unit is constituted by two anhydroglucose units that are linked by a β-1,4 glycosidic bond. Cellulose chains assemble via complex inter- and intramolecular H-bonding into crystalline structures [12, 13]. Crystalline sheets pack in a parallel fashion, building up filiform structures that can be isolated from the native material as cellulose nanocrystals (CNCs), which are also referred to as nanocrystalline cellulose (NCC) or cellulose nanowhiskers (CNWs). These rod-shaped, high-aspect-ratio nanoparticles (HARN; aspect ratio = length/diameter ≥ 3 [14]) exhibit a diameter of 5–40 nm and a length that can vary from 100–500 nm, when derived from plant sources, or from 1–3 µm when extracted from tunicates or algae (Fig. 1) [15–19]. In plant-derived cellulose, CNCs are further integrated into longer fibers that are composed of amorphous and crystalline domains and are commonly referred to as cellulose nanofibrils (CNF), nanofibrillated cellulose (NFC) or microfibrillated cellulose (MFC) [15, 20]. Thus, deconstruction of the hierarchical structure of plant cellulose by mechanical treatment and/or enzymatic [21] or chemical [22] treatments permits the isolation of CNFs [23]. The degradation of cellulose pulp into CNCs is generally achieved by hydrolysis of the non-crystalline domains with mineral acids such as hydrochloric [18], sulfuric [9, 24] or phosphoric acid [25]. In the case of the latter two acids, a frequently observed side-reaction is the formation of sulfate or phosphate ester groups with the surface hydroxyl groups of nanocellulose. The degree of functionalization and the nature of the functional groups determine the charge density and thereby the dispersibility of nanocellulose in liquid solvents or polymer matrices. The presence of surface ester groups also negatively affects the thermal stability of the nanocellulose and may affect their toxicological behavior [26, 27]. Bacterial cellulose (BC) is produced by bacteria in the form of continuous fibers with a diameter of 3–8 nm, which assemble into macroscopic meshes of high purity and crystallinity [11, 28, 29].

The high degree of crystallinity and the uniaxial orientation of the polymer chains bestow CNCs with an extraordinarily high stiffness (120–168 GPa) and strength [30, 31]. Other attractive features include a low density, low cost, the renewable nature of the source, and biodegradability. The high density of surface hydroxyl groups allow CNCs to interact with another and also polymeric matrix materials via H-bonding, which promotes very efficient stress transfer and makes CNCs ideal candidates as reinforcing fillers for polymers [9, 32]. It was shown that the H-bonding interactions can be switched “off” on demand, i.e. by exposure to a competing hydrogen-bond forming agent, and this has enabled the fabrication of stimuli responsive materials whose stiffness can be changed over several orders of magnitude [9, 33, 34]. CNCs can further form lyotropic phases, display a high surface area, and the abundance of surface hydroxyl groups makes the chemical modification of the surface readily possible. All these features make CNCs and other nanocellulose types interesting for a broad range of new applications including, use as a reinforcing filler in polymer nanocomposites [35, 36], the basis for stimuli responsive materials [9, 37, 38], as a nucleating agent [39, 40], a carrier for the controlled delivery of molecules [41], biosensors [42], and a component of tissue engineering scaffolds [43, 44]. In addition, the substitution of microcrystalline cellulose, which has long been used as rheology modifier in food products and cosmetic formulations, and as an excipient in tablets, with nanocellulose types can be envisioned to bring significant benefits beyond those described above.

The commercial production of CNCs and NFC has recently been launched and a gross world product of $600 billion is expected by 2020 [45]. For example, based on the technology developed by FPInovations and under the supervision of Domtar (Domtar Coorporation, Montreal, Canada), CelluForce© built a semi-commercial facility in 2010 with a capacity to produce 1000 kg CNCs per day [46, 47], whilst Innventia© reported a production of 100 kg CNFs per day in 2011 [48]. Several other entities have in the meantime installed production facilities for CNFs and CNCs that expand these initial capacities. The manufacturing of final products such as coatings, packaging materials, composite materials, aerogels for insulation or water filtration containing different types of nanocellulose has already commenced [49, 50]. Given these developments, the potential human health risks associated with exposure to these nanomaterials, especially in the form of respirable nanofibers as either a final product (e.g. in food and health care products), after extraction from a more complex material (e.g. after aging and degradation of a polymer nanocomposite or mechanical treatment of the latter), or at production or processing facilities (e.g. occupational exposure) must be understood [51, 52]. This is considered for all main portals of entry to the human body, including the skin, gastrointestinal tract, systemic circulation, and arguably, the most important, the lung [53]. The latter is considered the primary route of exposure to humans for any nanoparticle released into the environment (including, and especially, an occupational scenario) [54].

Since the first findings regarding the adverse biological impact of HARN, and their potential association with lung diseases were identified [55], special attention is being paid to the toxicology of engineered nanofibers [56]. The most prominently known fact surrounding fibers, is that exposure to asbestos fibers was associated with the development of epidemic lung disease states such as fibrosis, asbestosis, lung cancer, mesothelioma and pleural plaques [57]. Further studies on the toxicology of synthetic vitreous fibers (SVF), which are a group of inorganic materials containing aluminum or calcium silicates, led to the development of the fiber pathogenicity paradigm [58–60]. The *fiber paradigm* states that the length of a fiber is a key parameter that impacts the ability of a macrophage to phagocytize it; this results in frustrated phagocytosis [58], subsequent stimulation of inflammatory factors leading towards potential fibrosis or carcinogenic effects if the fiber is too long. However, the length is not the unique parameter involved in the toxicology of fibers; indeed the biopersistence of a fiber has been specifically identified as the key factor governing the biological response following (chronic) exposure [58, 61].

The fiber paradigm therefore highlights the importance of the form, shape and biological interaction of a substance when brought into contact with mammalian cells/tissue(s). Based on this understanding, and with the development of a disease commonly referred to as ‘brown lung’, observed in workers of the cotton industry exposed to cotton dust [62–64], several studies investigated the possible health risks associated with cellulosic materials. Tatrai et al. [65] administrated a single dose intratracheally (15 mg) of either cellulose powder, pine wood dust or a fiber-free extract from the same wood dust and observed after one month following exposure, granulomatous inflammation, fibrosis and alveobronchiolitis in vivo. The authors also observed in microscopic studies the presence of birefringent fibrous structures in the cytoplasm of formed multinucleated giant cells. However, these effects were not observed in fiber-free samples. In addition, other parameters such as the biopersistence of cellulose have been evaluated in several studies in vivo [66, 67] and in vitro [68]. Davis [67] reported in a 28-day inhalation study with rats the formation of alveolitis and granulomata. By contrast, a further in vivo study conducted by Warheit et al. [66]. that involved a 2-week inhalation period, no significant pulmonary effects were detected 3 months post exposure following exposure to microcellulose. Nevertheless, the authors reported the extremely limited rate of clearance of the fibers from the lungs of the animals which, as mentioned before, is an important parameter in fiber toxicology. Muhle et al. [69] also conducted an in vivo study and reported, after one year of exposure, a higher durability of cellulose fibers in the lung of rats (2 mg dose intratracheally) than chrysotile, a common form of asbestos. The biopersistence of cellulose nanofibers was also assessed in vitro using artificial lung airway lining fluid and macrophage phagolysosomal fluid, further supporting the durability of cellulosic fibers in a biological environment [68]. In light of these findings, and in further consideration of the differences between bulk and nanoscale materials, there is an imperative need to understand the potential hazard posed by nanocellulose, due to its nanoscale (1–100 nm) dimensions [53]. As a result, a number of studies have recently been conducted to shed light on this aspect. The objective of the present review is to summarize and critically discuss this recent work, and elucidate which key indicators can be utilized in the future in order to safely apply nanocelluose in different industries. It is important to note, that the discussion centered around this review is based upon the raw form of nanocellulose, and not that already applied in e.g. a polymer matrix. For a comprehensive review on applied forms of nanocellulose, please refer to [5].

### Life-cycle of nanocellulose

**Fig. 2:**
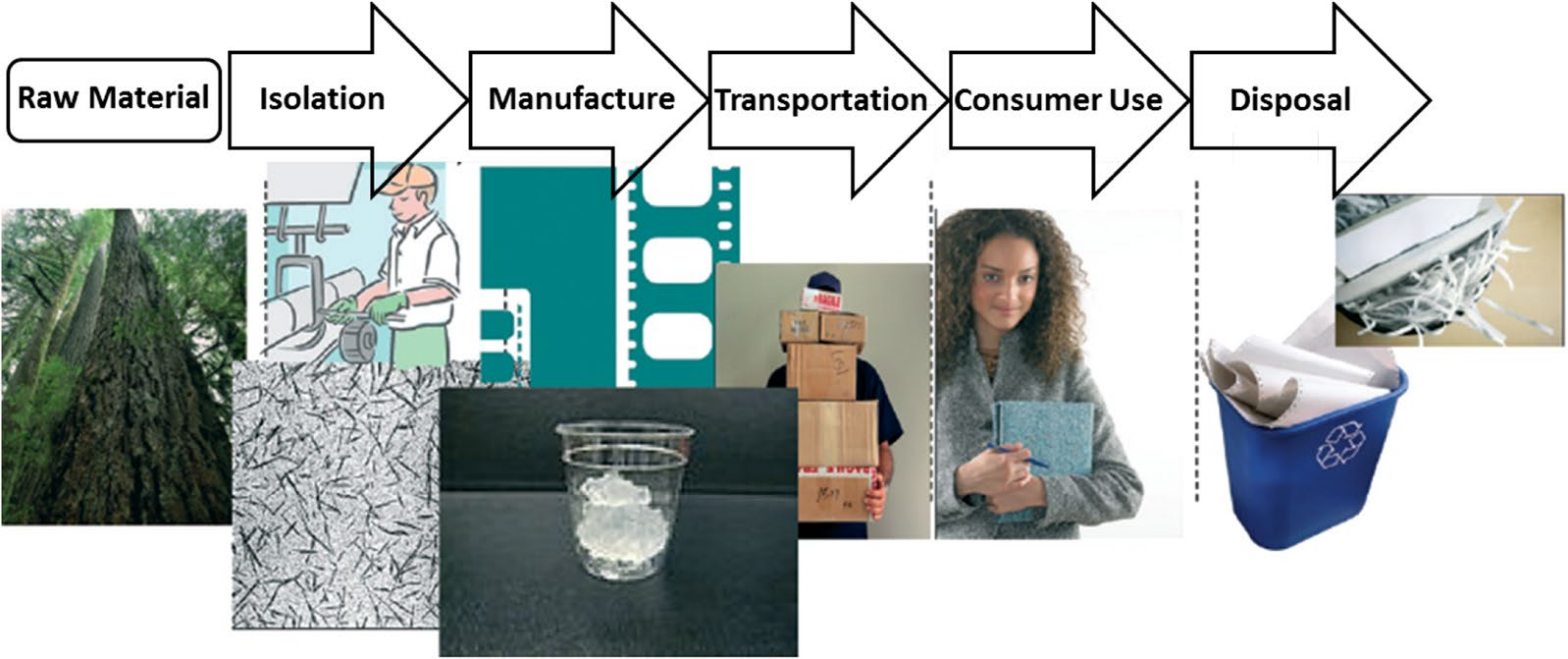
Life cycle of nanocellulose based composite materials where 5 different stages can be identified: production of raw materials or isolation (Stage 1), manufacture (Stage 2), transportation (Stage 3), consumer use (Stage 4) and disposal (Stage 5). Adapted from Shatkin et al. [70], with permission of The Royal Society of Chemistry

In order to evaluate the potential risk of any form of nanocellulose towards human health and the environment, its life-cycle must be studied in order to identify and analyze possible high- and low-risk scenarios. During the life-cycle of any manufactured nanomaterial, and product containing nanomaterials, several stages can be identified (Fig. 2): production of raw materials (Stage 1), manufacture (Stage 2), transportation (Stage 3), consumer use (Stage 4) and disposal (Stage 5). In a new life-cycle risk assessment framework (NANO LCRA) proposed by Shatkin and Kim [70], the different exposure scenarios during the life-cycle of nanocellulose in food packaging were evaluated and ranked as a function of the potential, magnitude, likelihood and frequency of the hazard. The authors identified the top four exposure scenarios to be (1) inhalation of dry, raw material by a facility employee during production, (2) application of dry, raw nanocellulose to create a film and inhalation during manufacturing, (3) inhalation of dry, raw nanocellulose powder during mixing with other materials to manufacture a product, and (4) inhalation by incidental contact with the raw form of nanocellulose. It has to be noted that transportation was not considered during evaluation of the life-cycle and that the risk assessment was performed for a specific application of nanocellulose, e.g. construction materials. However, analysis of the data suggests that the main exposure route would be the inhalation of (raw) nanocelluose, in whatever form, within an occupational setting. It is also important to note that the first exposure scenario at a consumer level appeared in tenth position, notably as the inhalation of sprayed wet nanoparticles [70]. It must be emphasized, however, that for other applications, such as the production of reinforced materials or the use as a food additive for example, other factors would have to be taken into consideration. In the case of polymer nanocomposites, for example, the release and inhalation of cellulose/polymer particles during processing steps such as drilling, cutting, and sanding, might be a concern [71]. Moreover, for many applications such as uses in healthcare products, cellulose might be surface functionalized, imparting new properties to the material and possibly triggering the need of an independent case study [72, 73].

Although first studies suggest that the inhalation of raw CNCs or CNFs would be the main exposure route for humans, little is known about the exposure concentrations or doses [74]. These parameters will strongly depend on each scenario, i.e., exposure concentrations in occupational activities are likely to be higher than those in consumer applications. Vartiainen et al. [75] measured the occupational exposure during grinding and spray-drying activities in a CNF production pilot plant. Under normal working conditions, e.g., with the grinding device placed inside a fume hood, the measured particle concentration in the air was as low as <4.000 particles/cm^3^ with some peaks reaching >8.000 particles/cm^3^. When the measurement was carried out inside the fume hood, the measured particle concentration reached 41.000 particles/cm^3^ with 75% of particles ranging between 10 and 30 nm in diameter. Similarly, during spray-drying the average particle concentration near the instrument was <10.000 particles/cm^3^ with a particle diameter between 20 and 60 nm. These findings suggest that humans can be readily exposed to nanocellulose in a variety of occupational settings at heightened concentrations. Nonetheless, understanding of the impact of chronic, repeated exposure to these airborne concentrations to human health however, remains, at best, limited.

### Biological impact of nanocellulose

Since human exposure, and to a lesser extent based on the current understanding, environmental exposure, to nanocellulose has been shown to be of a significant increase to normal airborne particle concentrations [75], and further to the concerns surrounding the potential hazard associated with HARN and nanomaterials in general [58], understanding of the structure–activity relationship of nanocellulose is vital. The purpose of the remainder of this review therefore, is to provide a critical overview of research directed towards exploring the biological impact and potential hazard of nanocellulose. An overview of key studies is provided in Table 1. In Table 1, together with the physical characteristics of the nanocellulose investigated, a description of the test system utilized, as well as the results of tests designed to assess cytotoxicity, (pro-)inflammatory response following nanocellulose exposure, the oxidative stress status of the biological system studied, as well as the potential for nanocellulose to elicit genotoxicity. Throughout the particle and fiber toxicology field, these endpoints are recognized as the most important drivers of nanomaterial toxicity [54]. For convenience, Table 1 provides a brief summary of the overall conclusions from each of these studies, although it is acknowledged that in some cases the entries may be overly simplified. It is important to further highlight that the biological systems highlighted through the main text and in Table 1 cover both in vitro, in vivo and ecosystem orientated models. This is a considered approach to convey the current understanding of the biological impact of raw nanocellulose, and its varying forms (which also change study-by-study) in terms of the biological response measured.

**Table 1:** Succinct overview of the key findings regarding the biological impact of nanocellullose samples studied within the literature

First author	Year	Ref	Nano-cellulose type	Dimensions	Test system	Cytotoxicity	Inflammatory response	Oxidative stress	Genotoxicity	Main conclusions from study
Moreira	2009	[82]	BC	50–1500 × 3–5 nm	3T3 fibroblasts, Chinese Hamster ovary cells	–	n/a	n/a	–	Benign material, beware of material modifications
Kovacs	2010	[76]	NCC	200 × 10 × 5 nm	Rainbow trout, *Daphnia magna, Ceriodaphnia dubia,* Fathead minnow, *Vibrio fischeri, Pseudokirchneriella subcapitata, Hydra attenuate, Danio rerio,* Rainbow trout hepatocyte cells	–	n/a	n/a	–	Low toxicity potential and low environmental risk
Jeong	2010	[81]	BC	n/a	Human umbilical vein epithelial cells; C57/Bl6 mouse model	–	n/a	n/a	n/a	Suitability for tissue engineering
Mahmoud	2010	[83]	CNC	130–200 × 10–20 nm	Human embryonic kidney cells (HEK 293) and *Spodoptera frugiperda* Ovary cells (Sf9)	+	n/a	n/a	n/a	Surface charge influences toxicity and uptake
Clift	2011	[91]	CNC	220 ± 6.7 × 15 ± 5 nm	3D Co-culture (A549 epithelial cells, combined with human blood monocyte derived macrophages (MDM) and dendritic cells (MDDC))	–	+	n/a	n/a	Length, stiffness and possibly origin affect CNC-cell interactions
Male	2012	[77]	NCC	120–140 × 3–6 nm	Chinese Hamster lung cells (V79) and *Spodoptera frugiperda* ovary cells (Sf9)	–	n/a	n/a	n/a	Origin/extraction, treatment and carboxylic acid content influence toxicity
Dong	2012	[78]	CNC	181 ± 9 × 5.0 ± 0.2 nm	Human brain microvascular endothelial cells (HBMEC), mouse endothelial brain cells (bEnd.3), RAW 264.7 macrophages, human breast epithelial cells (MCF-10A,MDA-MB-231 and MDA-MB-468), human hepatocyte cells (KB), prostate cancer cells (PC-3), Rat brain fibroblasts (C6)	–	n/a	n/a	n/a	Low unspecific uptake, no cytotoxicity appropriate for biomedical applications
de Lima	2012	[87]	CNF	White: 135 ± 50 × 14 ± 4 nm, Brown: 140 ± 45 × 11 ± 3 nm, Green: 180 ± 45 × 13 ± 2 nm, Ruby: 130 ± 25 × 10 ± 4 nm Curaua: 80**–**170 × 6–10 nm	*Allium cepa*, 3T3 fibroblasts, and lymphocytes	+	n/a	n/a	+	Genotoxicity depends on cell type and color used
Hannukainen	2012	[93]	NFC	n/a	BEAS 2B epithelial cells	–	n/a	–	+	Elucidation of limited genotoxicity
Pereira	2013	[88]	CNF	85–225 µm × 6–18 nm	Bovine fibroblasts	+	n/a	+	n/a	High dose of CNF exposure leads to negative cell effects
Pereira	2014	[84]	CNF	85–225 µm × 6–18 nm	*Chlorella vulgaris*	+	n/a	+	n/a	CNF exposure can affect algal viability and growth
Endes	2014	[80]	CNC	Cotton: 170 ± 72 × 19 ± 7 nm Tunicate: 2.3 ± 1.4 µm × 31 ± 7 nm	3D Co-culture [A549 epithelial cells, combined with human blood monocyte derived macrophages (MDM) and dendritic cells (MDDC)]	–	–	–	n/a	Benign nature of CNCs, independent of their dimensions
Hanif	2014	[86]	CNC	256 ± 64.8 nm 140.5 ± 37.5 nm 108.4 ± 94.8 nm 1174 ± 338.7 nm	3T3 fibroblasts and human colon epithelial cells (HCT116)	+ +	n/a	n/a	n/a	Cytotoxicity observed for concentrations below 250 µg/mL, dimensions irrelevant
Catalan	2014	[85]	CNC	135 ± 5 × 7.3 ± 0.2 nm	BEAS 2B epithelial cells and human blood monocyte derived macrophages		–	n/a	–	55% cytotoxicity mainly ≥100 µg/mL
Yanamala	2014	[90]	CNC	90.19 ± 3.03 nm 207.9 ± 49 nm n/a	C57BL/6 mouse model	+	+	+	n/a	Nanocellulose dimensions rather than the source exert a strong influence on the biological response
Stefaniak	2014	[68]	CNC, CNF	~105 × 10 nm, ~165 × 11 nm	RAW 264.7 macrophages	n/a	n/a	Cell free + in vitro –	n/a	High biodurability
Endes	2015	[79]	CNC	Cotton: 237 ± 118 × 29 ± 13 nm Tunicate: 2244 ± 1687 × 30 ± 8 nm	3D Co-culture [A549 epithelial cells, combined with human blood monocyte derived macrophages (MDM) and dendritic cells (MDDC)] @ Air–Liquid Interface	–	n/a	n/a	n/a	Length and concentration have a significant effect on CNC-cell interactions
Colic	2015	[89]	CNF	33 ± 2.5 µm × 10–70 nm	Mouse fibroblasts (L929), thymocytes, and peripheral blood mononuclear cells (PBMCs)	+	+	–	n/a	High concentration leads to observed effects
Shvedova	2016	[94]	CNC	158 ± 97 nm × 54 ± 17 nm	C57BL/6 mouse model	+	+	+	+	Male mice exhibit significantly higher adverse pulmonary effects compared to female mice (gender differences)
Farcas	2016	[95]	CNC	158 ± 97 nm × 54 ± 17 nm	*Cauda epididymal* sperm samples	+	+	+	+	Pulmonary exposure of CNC affects male mice reproduction system

These are structured, as referred to in the main text, as to the main biochemical endpoints studied within the field, including, *Cytotoxicity*, *Inflammatory Response*, Oxidative Stress and Genotoxicity. For each endpoint, +  response was observed and –  no response observed; n/a  not investigated). The final column highlights a brief, considered statement of the outcome of the referenced study. Studies are presented in the chronological order that they were published into the public domain

#### Cytotoxicity

One of the first important studies regarding the ecotoxicological impact of cellulose nanocrystals derived from ‘kraft pulp’ (CNC dimensions: 200 × 10 × 5 nm) was published by Kovacs et al. in 2010 [76]. The authors presented results from a realistic exposure scenario, i.e., suspension experiments with relevant dose ranges (0.03–10 g/L), that were based on the potential effluent in the vicinity of a CNC production site. The study included aquatic organisms from all trophic levels from bacteria, algae, crustacean, cnidarian to fish and investigated acute lethality (LC_50_ = the lethal concentration that reduces the biological system population to 50% viability), reproduction, growth, morphology, embryo development and cytotoxicity. Taking all results into consideration, the authors summarized the outcome as “non-concerning”.

**Fig. 3:**
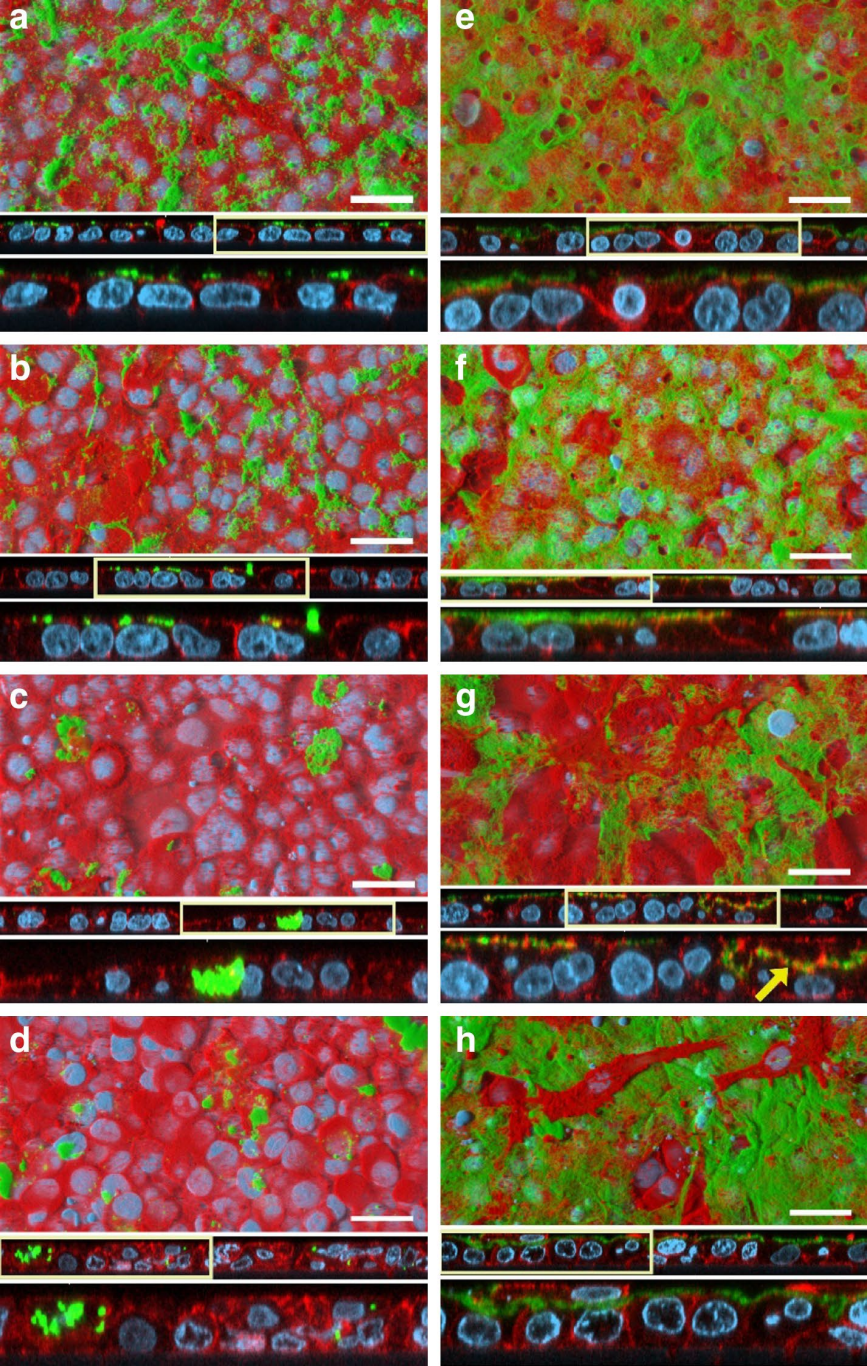
Length dependent clearance of CNCs by macrophages. Confocal laser scanning microscopy images of the triple-cell co-culture model exposed to 0.56 ± 0.25 μg/cm^2^ rhodamine-labeled CNCs isolated from cotton (*green* **a**–**d**) or 0.67 ± 0.09 μg/cm^2^ CNCs isolated from tunicates (**e**–**h**) via the ALICE system. Co-cultures were either immediately fixed (**a**, **e**) or after 1 (**b**, **f**), 24 (**c**, **g**), or 48 h (**d**, **h**) post exposure and stained for cytoskeleton (*red*) and nuclei (*cyan*). Images are presented as surface rendering (*top*), xz-projection of the z-stacks (*middle*), or twofold optical zoom (*bottom*). *Boxes* indicate digitally enlarged (×2) areas. *Arrow* shows fiber-F-actin interactions. *Scale bars* 30 μm. Reprinted with permission from Endes et al. [79] © 2015 American Chemical Society

Further to this, several studies on cellulose-human interactions confirmed the limited toxic potential of nanocellulose in terms of cytotoxicity in various experimental systems [77, 78]. A sophisticated triple-cell co-culture model of the human epithelial tissue barrier (formulated of a layer of epithelial cells, complimented by human blood monocyte derived macrophages and dendritic cells on the apical and basolateral sides respectively) was used in a study that showed no significant cytotoxicity of two different CNC types isolated from cotton (170 ± 72 × 19 ± 7 nm) and tunicates (2.3 ± 1.4 µm × 31 ± 7 nm) that were deposited onto the cells in realistic doses (0.14 ± 0.04, 0.81 ± 0.03 and 1.57 ± 0.03 µg/cm^2^) from aerosolized water-based suspensions [79, 80]. However, clearance, albeit based upon a dose, time and CNC-dependent manner, of deposited CNCs by macrophages was observed when cells were exposed to both of these types CNCs, with a lower efficiency associated with the tunicate CNCs (Fig. 3) [79]. Jeong and co-workers used bacterial cellulose (BC; no dimensions given [81]) in in vitro experiments with human umbilical vein endothelial cells (HUVECs) [81]. Neither of their experiments measuring cytotoxicity via the MTT assay, observing the morphology with light microscopy or assessing apoptosis/necrosis (Annexin V/Propidium Iodide staining) and cell-cycle via flow cytometry, showed significant altered outcomes after 24 or 48 h towards the exposure to high BC concentrations (0.1–1 mg/mL) compared to the negative control. Furthermore, in vivo exposure of 0.5–5 mg/mL BC administered via intraperitoneal injection to C57/Bl6 male mice showed no adverse effects after 7 days in comparison to sham exposures. Similar results with BC (50–1500 × 3–5 nm) were obtained by Moreira et al. [82] who could not detect significant changes in morphology or proliferation rates of mouse fibroblasts (3T3) and Chinese hamster ovary cells (CHO) in exposures ranging from 0.1–1 mg/mL.

However, there are also studies that have shown cytotoxic effects upon exposure to nanocellulose. Mahmoud and co-workers investigated uptake and membrane integrity in human embryonic kidney cells (HEK 293) and Sf9 insect cells and found that exposure to 0.1 mg/mL of negatively charged CNCs (ζ-potential −46.4 mV), which had been isolated from enzyme treated flax fibers (130–200 × 10–20 nm) and labeled with FITC (fluorescein isothiocyanate), led to membrane rupture under physiological pH in contrast to exposure to positively charged, RBITC-labeled (rhodamine B isothiocyanate) CNCs (ζ-potential 8.7 mV) [83]. Similar cytotoxic reactions were also reported using typical CNCs in exposures to algae [84] or bronchial cells (BEAS 2B) [85]. However, in both studies extremely high nanocellulose concentrations in respect to mammalian cell culture (0.25–5 mg/mL) were used [86–88]. Of note in this regard is the study by Colic and co-authors [89], who showed that only the exposure to extremely high concentrations of long, entangled cellulose nanofibrils (33 ± 2.5 µm × 10–10 nm; 0.25–1 mg/mL), the highest one covering the L929 monolayers almost completely, lead to impaired metabolic activity and reduced cell proliferation [89]. Furthermore in vivo, Yanamala measured elevated cytotoxicity (as determined by an increase in the activity of the enzyme lactate dehydrogenase) after the aspiration of wood pulp derived CNCs in mice (50, 100 and 200 μg/mouse), detecting similar strong reactions in the context of cytotoxicity compared to asbestos aspiration (50 μg/mouse) [90].

Overall, the incidence of benign results in terms of cytotoxicity, viability and impact upon mammalian cell morphology seems to be prevalent in the current literature upon the risk of nanocellulose. Despite this, the existence of adverse effects observed following nanocellulose exposure has to be taken into consideration when evaluating the total hazard posed by this material. Summarizing, single, low doses administration of nanocelluloses hint at the non-hazardous nature of nanocellulose, yet lack a degree of realism when considering human exposure. The importance of relevant exposure systems (cell type), dose, nanocellulose type/treatment/origin together with a clear material characterization is especially highlighted by the seemingly directly opposing results obtained by Mahmoud and co-authors (0.1 mg/mL FITC-labeled CNCs elicit cytotoxicity in human embryonic kidney cells (HEK 293) ovary cells (Sf9)) [83] *vs*. Dong et al. (0.01–0.05 mg/mL FITC-labeled CNCs induce no measurable cytotoxicity in a wide range of barrier and immune cell types in vitro) [78].

#### Inflammation

One of the key aspects of the nanoparticle-cell interaction is the potential for nanoparticles to elucidate a (pro-)inflammatory response from the cellular system being studied. In a realistic in vitro model of the human epithelial tissue barrier, it has been demonstrated that the exposure to CNCs does not induce a significant amount of (pro-)inflammatory mediators tumor necrosis factor-α (TNF-α) and interleukin-8 (IL-8), in contrast to asbestos fibers [91, 80]. The latter study [80] involved CNCs isolated from cotton (170 ± 72 × 19 ± 7 nm) and tunicates (2.3 ± 1.4 µm × 31 ± 7 nm) that were applied via nebulizing aqueous suspensions at a concentration range from 0.14 ± 0.04 to 1.57 ± 0.03 µg/cm^2^ by an air–liquid exposure approach. These results are underpinned by a study of Catalan et al., who exposed monocyte derived macrophage monocultures to 30–300 µg/mL cotton CNCs (135 ± 5 × 7.3 ± 0.2 nm) with no detection of TNF-α and IL-1β in comparison to microcrystalline cellulose (CNC aggregates that were micron-sized) [92]. Interestingly, Colic and co-workers showed an anti-inflammatory influence of cellulose nanofibril exposures on PBMCs (peripheral blood mononuclear cells) in vitro, as measured by downregulation of IL-2, IFN-γ (interferon-γ) and IL-17, of, which was only observed at considered high doses (0.25–1 mg/mL) [89]. However, Clift et al. (220 ± 6.7 × 15 ± 5 nm) [91], who used the same 3D triple-cell co-culture model of the human epithelial tissue barrier highlighted above and applied CNCs via aqueous suspensions, showed an increase in IL-8 response when exposed to 30 µg/mL cotton CNCs. An extensive screening study by Yanamala and colleagues that explored the administration of CNCs after different processing steps (wood pulp CNCs applied as isolated in suspension and kept in suspension vs. isolated and freeze dried to powder before re-suspension) found that both preparations of CNCs have the potential to induce inflammatory effects following pharyngeal aspiration in mice [90]. The authors detected significantly elevated pulmonary influxes of total cells, especially PBMCs compared to negative controls and mice exposed to asbestos. Increased expression of cytokines (IL-1α, IL-1β, IL-5, IL-6, IL-12 p40, G-CSF, GM-CSF, KC, MCP-1, MIP-1α, MIP-1β, and TNF-α) involved in acute inflammatory reactions compared to the control could be detected. Interestingly, depending on the pre-treatment from which the CNCs were applied, either a T-helper cell subtype 1 (Th1) mediated immune response (freeze dried before resuspension) or the induction of a Th2 associated response (only suspension) could be observed.

Despite the data discussed the above paragraph (Table 1), there remains a prominent lack of coherent data to substantially, and specifically evaluate the potential of nanocellulose to pose a relevant hazard towards human health via an inflammatory immune response. Nevertheless, the existing studies point out that the physico-chemical characteristics, especially the aggregation status, of CNCs can have a (direct) detrimental impact towards elucidating a (pro-)inflammatory response [90]. Moreover, overload exposures often mask the underlying specific mechanisms of toxicity and can only point at a general direction of potential hazard. In terms of inflammation, especially the chronic or repeated low dose exposure as the most realistic scenario for human exposure must be focused upon in future research.

#### Oxidative stress

Little is known about the radical forming potential of nanocellulose in cell-free and cellular environments, with studies mainly reporting insignificant impact on the oxidative stress status of the cells unless extremely high concentrations are applied (cotton CNFs, 85–225 µm × 6–18 nm; 2–5 mg/mL, bovine fibroblasts), similar to the endpoints of cytotoxicity and inflammation [88]. Only a few studies include the measurement of radical oxygen species formation [68, 89], the activity of antioxidant enzymes such as superoxide dismutase (SOD) or peroxiredoxin [88], and the depletion of antioxidant peptides such as glutathione [80, 89]. Interestingly, Stefaniak et al. observed significantly increased radical formation (∙OH) by CNCs (~105 × 10 nm) and CNFs (~165 × 11 nm) in a cell free experiment in contrast to benchmark MCC (<10 µm × <2 µm) with absent, consecutive cellular reactions in macrophages [68]. These results are especially alarming as the study also revealed a high durability in artificial lung fluid. In summary, it has been commonly reported that no significant oxidative stress is evident in vitro following nanocellulose exposure, i.e. using cotton (170 ± 72 × 19 ± 7 nm) or tunicate (2.3 ± 1.4 µm × 31 ± 7 nm) CNCs (0.14 ± 0.04 − 1.57 ± 0.03 µg/cm^2^) in the human epithelial tissue barrier model previously described [80], nanofibrillated celluloses (9.5–950 µg/cm^2^) on bronchial cells (BEAS 2B) [93], CNFs in high dose experiments with bovine fibroblasts (85–225 µm × 6–18 nm; 2–5 mg/mL) [88] and CNFs in lower doses to human fibroblasts (L929; >10 µm × 10–35 nm; 31.5 µg/ml–1 mg/ml) [89]. However, measurable biological effects were shown by Pereira et al. as a slight increase in SOD activity in the algae *Chlorella vulgaris* after exposure to 1, 50 and 100 μg/mL cotton CNFs (85–225 μm × 6–18 nm) [88].

The oxidative stress status of a cell has a relevant influence most importantly in chronic exposures where it, together with its intrinsic biopersistence, can lead to severe damage and resulting disease as seen with other HARN materials [56]. The findings in cell-free experiments Stefaniak and colleagues [68] should point out the importance to substantiate the research in this direction regarding the potential adverse biological impact of nanocellulose.

#### Genotoxicity

In recent years the investigation of damage to or changes in the genetic information within a cell induced by nanoparticle exposure came into focus; including the measurement of DNA strand breaks, formation of micronuclei and the potential for mutagenicity. Only a few studies have so far investigated the genotoxic influence of nanocellulose. Although the typical dimensions of nanocellulose result in an unlikeliness of nuclear translocation however is not to be excluded without further evidence. Nevertheless, the hindrance of cell-division, viability or indirect genotoxicity has to be especially highlighted when surface functionalizations are used to alter the bare and so far benign surface of nanocellulose.

Of the studies pertinent to this biological endpoint regarding nanocellulose, no effects in terms of micronuclei formation could be observed with BEAS 2B cells at low concentrations of cotton CNCs (2.5–100 μg/mL; 135 ± 5 × 7.3 ± 0.2 nm) over 48 h [92]. Kovacs et al. reported no changes in DNA quality after exposures to up to 2 mg/mL kraft pulp CNCs (200 × 10 × 5 nm) in primary rainbow trout hepatocytes [76]. Similar results were obtained when CNCs isolated from BC (50–1500 × 3–5 nm) were used in a comet assay and the AMES test in a concentration of 0.1 – 1 mg/mL after 48 h [82]. However, 0.01–1% white, colored cotton and curaua nanofibers (white 135 ± 50 × 14 ± 4 nm, brown 140 ± 45 × 11 ± 3 nm, green 180 ± 45 × 13 ± 2 nm, ruby 130 ± 25 × 10 ± 4 nm and curaua: 80–170 × 6–10 nm) showed the ability to induce negative changes in the relative mitotic index and chromosomal aberration of *Allium cepa* cells as well as DNA strand breaks in concentrations of 0.1% of brown cotton and curaua fibers in animal cells (human lymphocytes, 3T3 mouse fibroblasts) [87]. Furthermore, Hannukainen et al. reported a potential genotoxic effect by the exposure of BEAS 2B epithelial cells to NFC (950 μg/cm^2^; 24 h) measured by the comet assay [93].

Finally, important recent research has shown that some CNCs are able to induce all four biological endpoints, highlighting that through complex cellular cascades, that all four biochemical processes can induce deleterious effects, albeit only in males in vivo. In recent studies by Shvedova et al., and Farcas et al., it has been shown that following pulmonary exposure of CNCs to C57BL/6 mice, that, after analysis over a chronic period, male mice were more susceptible to exhibit increased cytotoxicity, which was further associated with a heightened inflammatory and oxidative stress response compared to female mice. Further evidence was shown that these biochemical effects led to significant genotoxicity [94]. In a further study, a similar author team elucidated further that the genotoxic effects were highly detrimental to the male reproductive system [95].

## Summary

It is apparent from the research conducted regarding the potential hazard posed by various forms of nanocellulose, especially towards human and environmental health, that the current understanding of its structure–activity relationship is equivocal and incoherent. Whilst a multitude of studies show the overall benign nature of nanocellulose, others stress the potential for adverse effects (overview Table 1).

It appears that many of the observed differences can be attributed to the variation in cell systems, material origin, treatment and characterization, cell exposure doses reaching non-realistic concentrations of nanocellulose, exposure scenarios or the lack of thorough characterization of the administered CNCs and/or the biological systems used. Some studies focus on the inhalation route as one of the main entry portals for particulates in occupational settings [79, 80, 91, 92, 96]. Others focus on the reaction of immune cells as important drivers of toxicity [90]. Some of the observed cellular responses are the result of heavily overloaded systems and the outcomes, therefore, are deemed to be an effect of the dose and not the nanomaterials themselves [88]. So far, the approach of most of the experiments is a general hazard assessment with little regard to realistic exposure doses, particle characteristics during exposure, time frames or exposure scenarios. Additionally, due to the nature of nanocellulose it is challenging to track it during uptake and fate due to a lack of analytical methods feasible to measure nanocellulose in biological systems. Therefore, the morphological impact or organ distribution after exposure is limited. Nevertheless, the overall results could be interpreted that most of the studies hint at a limited hazard potential of nanocellulose. From the data highlighting a potential hazard associated with nanocellulose however, such possibilities can be circumvented or diminished by avoiding those nanocellulose types with extreme length (>5 µm), overload doses or in a physical format that induces biological adverse effects such as freeze-dried and re-suspended powder. It seems that the limiting factor in guiding the scientific output regarding nanocellulose toxicity is the lacking knowledge of incidence and in situ exposure doses as well as the specific types of nanocellulose mostly used, i.e. commercial products should be tested instead of in house products. Clear understanding of the specific physical and chemical properties of currently produced and used nanocellulose and realistic exposure doses is of the utmost importance and inevitable.

Finally, data in acute exposure scenarios reported upon the structure–activity relationship of nanocelluloses indicate that they do not pose as greater risk to human (and environment) health as other HARN currently being produced and potentially used in similar applications (e.g. CNTs). Until further results elucidate the potential of adverse health/environmental effects posed by nanocellulose, avoiding exposure with specialized personal protection gear and release is the best way for protection. Clarity must be obtained as to the health implications of low dose, chronic and repeated exposure to nanocellulose in its many different forms, as this holds the key to their potential advantageous use across a multitude of disciplines and applications.

## Abbreviations

ALI: air–liquid interface
BC: bacterial cellulose
CHO: Chinese Hamster Ovary cells
CNC: cellulose nanocrystal
CNF: cellulose nanofibril/fiber
CNT: carbon nanotube
CNW: cellulose nanowhisker
CSF: colony stimulating factor
FITC: fluorescein isothiocyanate
G-CSF: granulocyte-CSF
HARN: high aspect-ratio nanoparticles
HEK 293: human embryonic kidney cells
IL: interleukin
INF: interferon
KC: keratinocyte chemoattractant
LC_50_: lethal concentration (indicating 50% loss in viability)
LDH: lactate dehydrogenase
MCP: monocyte chemoattractant protein
MFC: microfibrillated cellulose
MIP: macrophage inflammatory protein
MTT: 3-(4,5-dimethylthiazol-2-yl)-2,5-diphenyltetrazolium bromide
NCC: nanocrystalline cellulose
NFC: nanofibrillated cellulose
PBMC: peripheral blood mononuclear cell
RBITC: rhodamine-B-isothiocyanate
Sf9: *Spodoptera frugiperda* ovary cells
SOD: superoxide dismutase
SVF: synthetic vitreous fibers
TNF: tumor necrosis factor
